# The Tri State Medical Society

**Published:** 1883-04

**Authors:** G. W. Burton

**Affiliations:** Sec’y.


### Society Reports.

### ARTICLE XIII.

### THE TRI-STATE MEDICAL SOCIETY.

One of the most important medical events of the year will be the meeting of the Tri-State Medical Society, at Indianapolis, in September. Already the work for this convention is far advanced, owing to the almost perfect organization of the society.

Some years ago the society found that the hospitality of the citizens, in the places of meeting, to some extent interfered with its proper work, and that long papers crowded out shorter and better ones. It was then resolved that the society accept of no banquets, etc., and that all papers be limited to twenty-five minutes.

From that time the increase in interest and attendance was marked, and now, during each of the three days of the meeting, three sessions are held, fully occupied with short, practical papers and discussions, the authors having been previously selected by the committee on programme.

The "Tri-State" is in exact harmony with the different State and other local societies, leaving to them all matters of legislation and ethics, and requiring that its members be also members in good standing of one or more of these.

The territory embraced is Indiana, Kentucky and Illinois, to which Cincinnati and St. Louis have been added. At the last meeting there were many visitors from other States.

The "Tri-State" holds front rank in reputation, in both Europe and America, as a working society, and work has been the secret of its success.

For further information, address any of the officers: Dr. Wm. Porter, St. Louis, president; Dr. G. W. Burton, Mitchell, Ind., secretary; Dr. F. W. Beard, Vincennes, Ind., treasurer; Dr. T. B. Harvey, Indianapolis, chairman of committee of arrangements; Dr. J. L. Thompson, Indianapolis, chairman of committee on programme.

Respectfully,

G. W. BURTON, *Sec'y.*

